# Supplementary material for: Conserved phenotype and function of human brain border-associated macrophages in iPSC-derived models
Source: bioRxiv. 2025 Dec 13:2025.12.11.693582. Preprint. [Version 1] doi: 10.64898/2025.12.11.693582 (PMC12713604; doi:10.64898/2025.12.11.693582)
Supplement: 1 [file NIHPP2025.12.11.693582V1-supplement-1.pdf]

bioRxiv preprint doi: https://doi.org/10.64898/2025.12.11.693582; this version posted December 14, 2025. The copyright holder for this preprint (which was not certified by peer review) is the author/funder, who has granted bioRxiv a license to display the preprint in perpetuity. It is made available under aCC-BY 4.0 International license.

SUPPLEMENTAL FIGURES

FIGURE S1

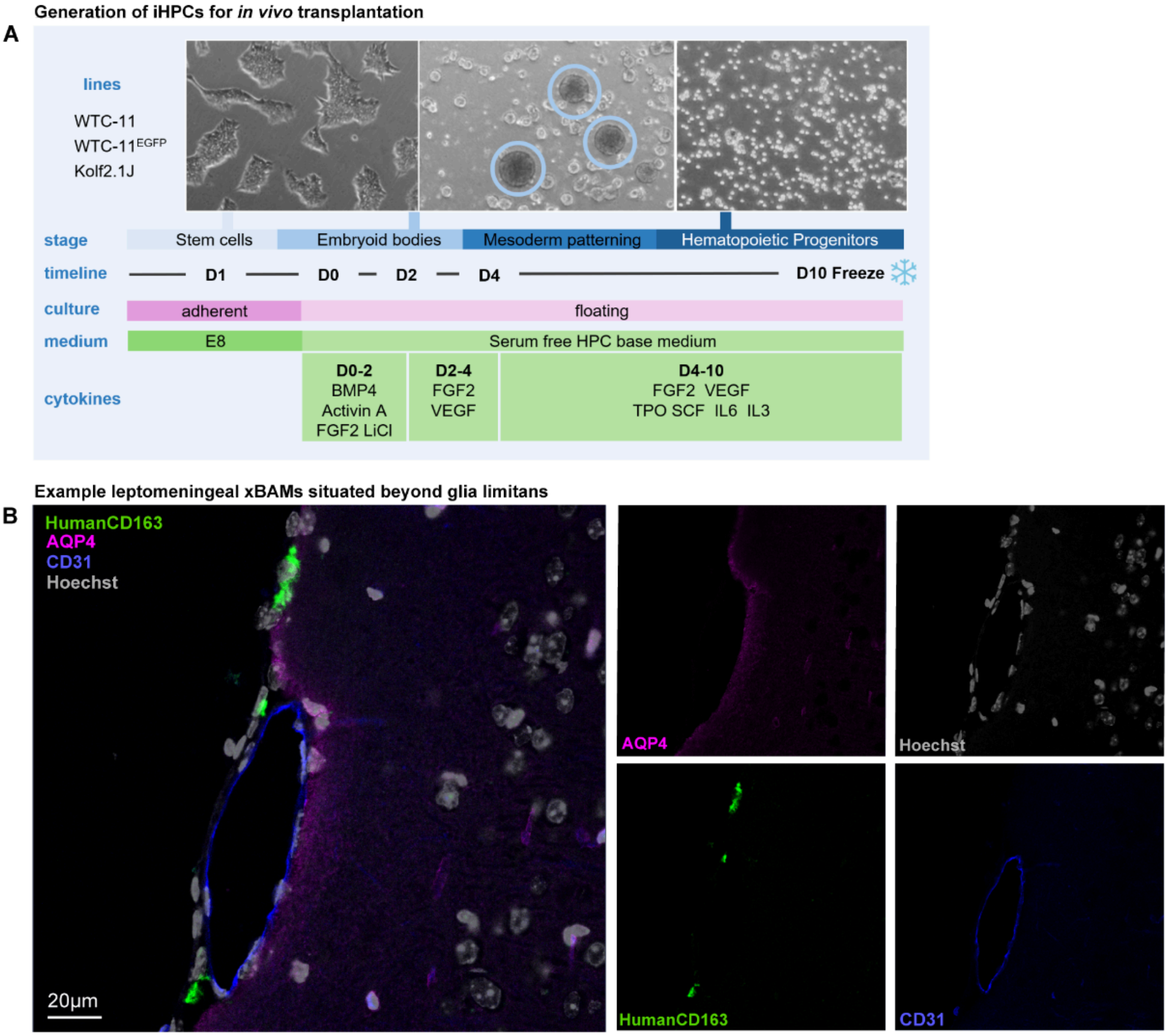

Figure S1. Further characterization of chimeric human-murine brain immune compartment

(**A**) Schematic diagram of experimental design for generating iPS-derived HPCs for transplantation (see **Methods**).

(**B**) Example image of human xBAM (labeled with anti-human CD163) located along a pial vessel (labeled with anti-mouse CD31) in the leptomeninges (delineated with anti-mouse AQP4).

bioRxiv preprint doi: https://doi.org/10.64898/2025.12.11.693582; this version posted December 14, 2025. The copyright holder for this preprint (which was not certified by peer review) is the author/funder, who has granted bioRxiv a license to display the preprint in perpetuity. It is made available under aCC-BY 4.0 International license.

FIGURE S2

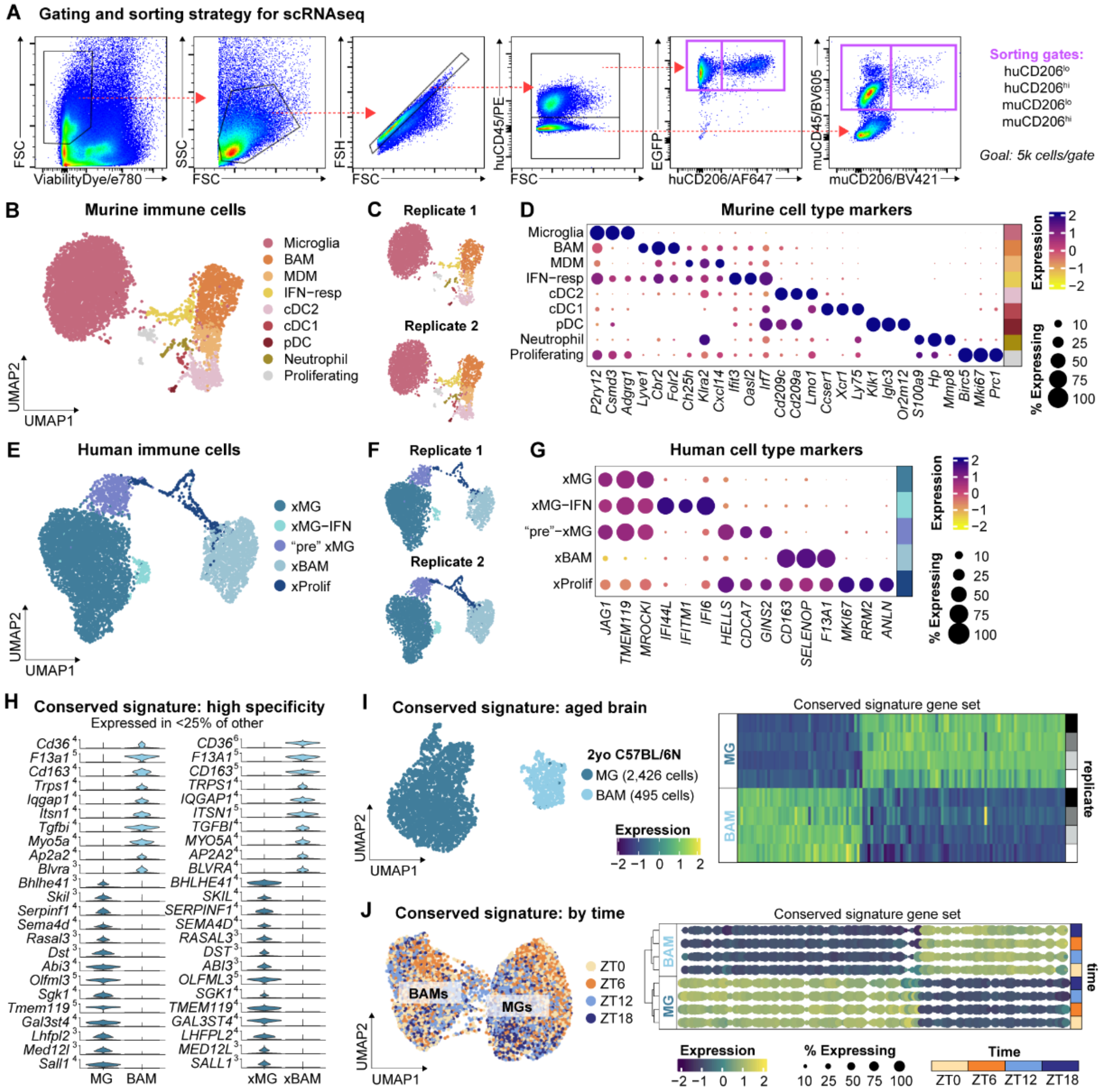

bioRxiv preprint doi: https://doi.org/10.64898/2025.12.11.693582; this version posted December 14, 2025. The copyright holder for this preprint (which was not certified by peer review) is the author/funder, who has granted bioRxiv a license to display the preprint in perpetuity. It is made available under aCC-BY 4.0 International license.

Figure S2. Further characterization of conserved BAM versus microglia signature

(**A**) Flow cytometry gating and sorting strategy for scRNAseq of chimeric immune compartment. Purple boxes represent sorting gates.

(**C-D**) UMAP of annotated murine immune cell subsets, UMAP split by biological replicate, and heat map of murine immune cell type markers. cDC1/2 = conventional dendritic cell type 1/2, pDC = plasmacytoid DC.

(**E-G**) UMAP of annotated human immune cell subsets, UMAP split by biological replicate, and heat map of human immune cell type markers.

(**H**) Violin plot of highest specificity BAM vs. microglia markers, indicated by conserved expression under 25% in the opposing cell type across both human and murine cells.

(**I**) UMAP of microglia and BAMs from 2 year-old C57BL/6 male mice (n of four biological replicates) and heatmap of expression of conserved signature gene set across cell types.

(**J**) UMAP of microglia and BAMs from 2.5 month-old C57BL/6 male mice (n of 12 biological replicates total, 3 replicates per time point) grouped by time and k-means clustering of cell types by time using conserved signature gene set. Regardless of time of day, hierarchical clustering still distinguishes BAMs and microglia using only the conserved signature gene set.

bioRxiv preprint doi: https://doi.org/10.64898/2025.12.11.693582; this version posted December 14, 2025. The copyright holder for this preprint (which was not certified by peer review) is the author/funder, who has granted bioRxiv a license to display the preprint in perpetuity. It is made available under aCC-BY 4.0 International license.

FIGURE S3

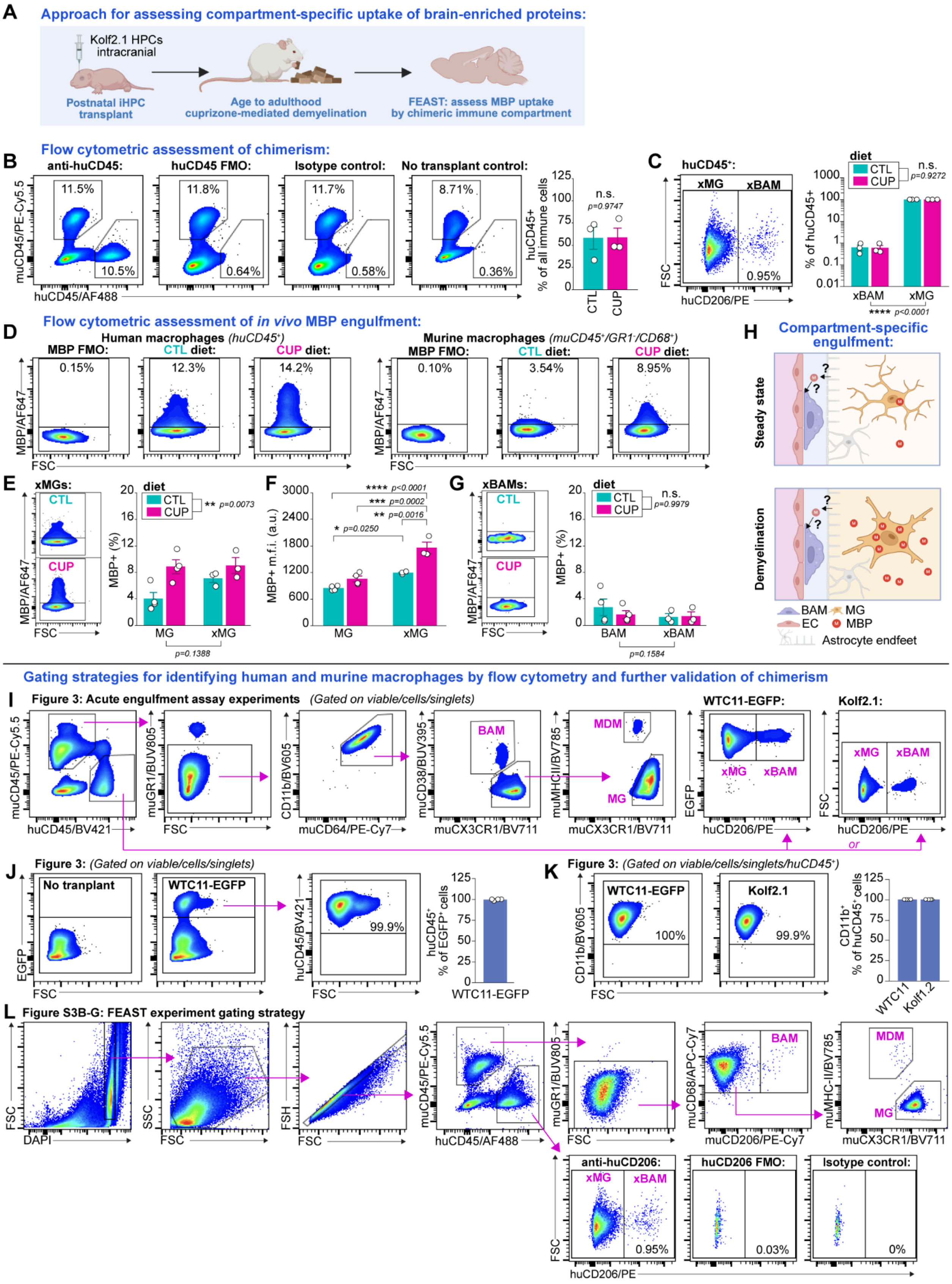

bioRxiv preprint doi: https://doi.org/10.64898/2025.12.11.693582; this version posted December 14, 2025. The copyright holder for this preprint (which was not certified by peer review) is the author/funder, who has granted bioRxiv a license to display the preprint in perpetuity. It is made available under aCC-BY 4.0 International license.

Figure S3. A flow cytometric toolkit for assessing *in vivo* function of human brain macrophages

(**A**) Schematic diagram of experimental design. Mice received a postnatal transplant of iPS-derived HPCs (Kolf2.1). In adulthood, mice underwent cuprizone-mediated demyelination and MBP uptake was assessed across the chimeric immune compartment via FEAST.

(**B**) Pseudo-colored plots demonstrating specificity of anti-human CD45 staining in chimeric brains and group-level quantification of chimerism across conditions. CTL = control diet, CUP = cuprizone diet. No effect of diet on chimerism (two-tailed student's t-test; n of 3 chimeras per diet).

(**C**) Pseudo-colored plots demonstrating specificity of anti-human CD206 staining in chimeric brains and group-level quantification of distribution of chimeric cell types across conditions. No effect of diet on composition of chimeric immune compartment (two-way ANOVA; main effects shown; diet*cell type interaction effect: p=0.8226; n of 3 chimeras per diet).

(**D**) Representative pseudo-colored plots of MBP uptake by macrophages of human and murine origin across control and cuprizone diets, with negative control MBP staining.

(**E and F**) Representative pseudo-colored plots of MBP signal in xMGs from control and cuprizone-treated mice, and group-level quantification of MBP-positivity and signal in MGs and xMGs across conditions. Main effect of diet but not cell type, and no interaction between the two (p=0.1813), on MBP-positivity, and cell type*diet interaction effect on MBP signal within MBP+ cells: p=0.0315. Quantifications using two-way ANOVAs, post-hoc testing with Tukey's HSD, n of 3-4 per condition.

(**G**) Representative pseudo-colored plots of MBP signal in xBAMs from control and cuprizone-treated mice. No effect of diet or cell type on MBP uptake by BAMs and xBAMs (two-way ANOVA; main effects shown; diet*cell type interaction effect: p=0.6086; n of 3-4 per condition).

(**H**) Schematic diagram of compartment-specific engulfment. Cuprizone-mediated demyelination induces robust increases in MBP uptake by MGs and xMGs but not BAMs/xBAMs.

(**I**) Gating strategy for acute *ex vivo* engulfment assays in Figure 3.

(**J**) Verification of fate of xenotransplanted human cells. Representative pseudo-colored plots of EGFP expression in WTC11-EGFP-transplanted mice, and huCD45 expression within EGFP$^+$ cells. The proportion of EGFP$^+$ cells that are huCD45$^+$ is not different from 100% (two-tailed one-sample t-test against mean of 100, p = 0.3163; n of 4 independent replicates). All xenotransplanted cells detected by flow cytometry had thus become CD45$^+$ leukocytes.

(**K**) Verification of identity of xenotransplanted human cells. Representative pseudo-colored plots of CD11b expression in huCD45$^+$ cells from WTC11-EGFP- and Kolf2.1-transplanted mice. 99.9±0.01% of

bioRxiv preprint doi: https://doi.org/10.64898/2025.12.11.693582; this version posted December 14, 2025. The copyright holder for this preprint (which was not certified by peer review) is the author/funder, who has granted bioRxiv a license to display the preprint in perpetuity. It is made available under aCC-BY 4.0 International license.

all huCD45$^+$ were CD11b$^+$ (mean±SEM, n of 4 per condition). All xenotransplanted leukocytes detected by flow cytometry had thus become CD11b$^+$ macrophages.

(**L**) Gating strategy for FEAST experiment in Figure S3B-G.

For all panels: points represent individual mice, bars and error bars represent mean and SEM. Illustrations made with BioRender.

bioRxiv preprint doi: https://doi.org/10.64898/2025.12.11.693582; this version posted December 14, 2025. The copyright holder for this preprint (which was not certified by peer review) is the author/funder, who has granted bioRxiv a license to display the preprint in perpetuity. It is made available under aCC-BY 4.0 International license.

SUPPLEMENTAL TABLES

TABLE S1

p_val	avg_log2FC	pct.1	pct.2	p_val_adj	cluster	gene
0	5.46962189	0.94	0.04	0	Microglia	GRCm39-Csmd3
0	5.66051576	0.87	0.03	0	Microglia	GRCm39-Adgrg1
0	5.5668271	0.83	0.03	0	Microglia	GRCm39-Gm3667
0	5.38300052	0.81	0.03	0	Microglia	GRCm39-Gm2629
0	5.58954782	0.71	0.02	0	Microglia	GRCm39-Gm3264
0	5.84623379	0.65	0.01	0	Microglia	GRCm39-Ecscr
0	5.76471541	0.61	0.02	0	Microglia	GRCm39-Gm3739
0	5.5182124	0.60	0.02	0	Microglia	GRCm39-Klf12
0	5.79740698	0.58	0.01	0	Microglia	GRCm39-Atp8a2
0	5.60294007	0.58	0.02	0	Microglia	GRCm39-Lag3
1.36E-279	5.69181065	0.52	0.01	5.62E-275	Microglia	GRCm39-Ifitm10
2.26E-232	6.06858971	0.45	0.01	9.37E-228	Microglia	GRCm39-Gm10406
5.87E-213	5.2969666	0.43	0.01	2.43E-208	Microglia	GRCm39-Npnt
2.88E-173	5.5469902	0.37	0.01	1.20E-168	Microglia	GRCm39-Cdh23
2.30E-158	5.57743288	0.34	0.01	9.54E-154	Microglia	GRCm39-Slco4a1
1.02E-147	6.21248718	0.32	0.01	4.22E-143	Microglia	GRCm39-Tspan18
2.47E-141	6.23558785	0.30	0.01	1.02E-136	Microglia	GRCm39-Zfp697
1.39E-134	5.48072365	0.30	0.01	5.76E-130	Microglia	GRCm39-Gm33699
4.20E-124	5.50061017	0.28	0.01	1.74E-119	Microglia	GRCm39-2610042L04Rik
3.33E-111	6.10579602	0.25	0.01	1.38E-106	Microglia	GRCm39-Gm2974
0	5.11837025	0.95	0.07	0	BAM	GRCm39-F13a1
0	5.57033378	0.88	0.03	0	BAM	GRCm39-Cbr2
0	5.86473602	0.63	0.02	0	BAM	GRCm39-Folr2
0	5.49185138	0.63	0.04	0	BAM	GRCm39-Cd163

bioRxiv preprint doi: https://doi.org/10.64898/2025.12.11.693582; this version posted December 14, 2025. The copyright holder for this preprint (which was not certified by peer review) is the author/funder, who has granted bioRxiv a license to display the preprint in perpetuity. It is made available under aCC-BY 4.0 International license.

0	5.03271116	0.55	0.03	0	BAM	GRCm39-Clec4n
0	5.9211732	0.52	0.01	0	BAM	GRCm39-Igf1
0	7.23723969	0.45	0.01	0	BAM	GRCm39-Lyve1
0	5.71068531	0.46	0.03	0	BAM	GRCm39-Ccl24
0	8.15707266	0.42	0.01	0	BAM	GRCm39-Fcna
0	5.35941943	0.41	0.01	0	BAM	GRCm39-Slc15a2
0	5.93891238	0.39	0.01	0	BAM	GRCm39-Gpx3
0	4.79228099	0.40	0.03	0	BAM	GRCm39-Cd36
0	5.84176046	0.38	0.01	0	BAM	GRCm39-Clmp
0	7.7288577	0.37	0.00	0	BAM	GRCm39-Tslp
0	7.08463085	0.34	0.00	0	BAM	GRCm39-Ednrb
0	5.78243339	0.32	0.01	0	BAM	GRCm39-Chp2
0	9.44938919	0.28	0.00	0	BAM	GRCm39-Cd209f
2.22E-296	6.04967594	0.30	0.01	9.21E-292	BAM	GRCm39-Egfl7
5.11E-253	5.21795048	0.26	0.01	2.12E-248	BAM	GRCm39-Pla2g2d
3.35E-249	6.38117168	0.26	0.01	1.39E-244	BAM	GRCm39-Cd38
4.06E-305	3.33259495	0.84	0.11	1.68E-300	MDM	GRCm39-H2-Ea
1.09E-295	3.1397676	0.86	0.11	4.50E-291	MDM	GRCm39-H2-Aa
6.43E-292	3.21838066	0.91	0.14	2.67E-287	MDM	GRCm39-Cd74
2.54E-290	3.1257154	0.83	0.11	1.05E-285	MDM	GRCm39-H2-Eb1
8.60E-264	2.92208601	0.93	0.17	3.56E-259	MDM	GRCm39-Fxyd5
1.84E-220	3.04946172	0.86	0.18	7.64E-216	MDM	GRCm39-H2-Ab1
5.41E-200	2.70625815	0.65	0.09	2.24E-195	MDM	GRCm39-Ccr2
1.09E-176	2.83141273	0.78	0.18	4.53E-172	MDM	GRCm39-H2-DMb2
7.26E-157	3.16674356	0.56	0.09	3.01E-152	MDM	GRCm39-H2-DMb1
5.69E-154	3.99443035	0.38	0.04	2.36E-149	MDM	GRCm39-Cxcl14
1.85E-138	3.39183907	0.43	0.06	7.68E-134	MDM	GRCm39-Klra2
7.61E-103	4.20790964	0.25	0.02	3.15E-98	MDM	GRCm39-Ch25h

bioRxiv preprint doi: https://doi.org/10.64898/2025.12.11.693582; this version posted December 14, 2025. The copyright holder for this preprint (which was not certified by peer review) is the author/funder, who has granted bioRxiv a license to display the preprint in perpetuity. It is made available under aCC-BY 4.0 International license.

3.21E-93	4.09908952	0.33	0.05	1.33E-88	MDM	GRCm39-Spp1
1.71E-90	2.53864082	0.40	0.07	7.09E-86	MDM	GRCm39-Clec4b1
2.29E-87	3.04789881	0.31	0.05	9.48E-83	MDM	GRCm39-Sirpb1c
2.25E-82	2.85093751	0.46	0.11	9.34E-78	MDM	GRCm39-Cd72
1.26E-71	2.69408381	0.35	0.07	5.23E-67	MDM	GRCm39-Lilrb4b
1.53E-57	2.68819674	0.29	0.06	6.33E-53	MDM	GRCm39-Ciita
2.49E-42	2.61975849	0.25	0.06	1.03E-37	MDM	GRCm39-B3gnt8
3.70E-37	2.5558484	0.26	0.07	1.53E-32	MDM	GRCm39-Slc1a5
0	5.9821722	0.93	0.03	0	cDC2	GRCm39-S100a4
0	5.97869132	0.93	0.04	0	cDC2	GRCm39-Olfm1
0	6.89181674	0.77	0.02	0	cDC2	GRCm39-Cd209c
0	6.81904574	0.66	0.01	0	cDC2	GRCm39-Cd209a
0	7.08884311	0.64	0.01	0	cDC2	GRCm39-Lmo1
0	5.90948984	0.63	0.01	0	cDC2	GRCm39-Slamf7
0	6.27799801	0.58	0.01	0	cDC2	GRCm39-Tnip3
0	6.18560431	0.53	0.01	0	cDC2	GRCm39-Klra17
0	7.62115658	0.53	0.01	0	cDC2	GRCm39-Ear2
0	6.19202994	0.50	0.01	0	cDC2	GRCm39-Fcor
0	6.64765379	0.46	0.01	0	cDC2	GRCm39-Klrd1
0	7.64693578	0.46	0.00	0	cDC2	GRCm39-Kcne3
0	7.0481555	0.41	0.00	0	cDC2	GRCm39-Mmp12
0	6.29586419	0.40	0.01	0	cDC2	GRCm39-Klrb1b
0	6.10151355	0.39	0.01	0	cDC2	GRCm39-Grap2
0	9.47230863	0.30	0.00	0	cDC2	GRCm39-Ccdc170
3.23E-302	5.91637547	0.33	0.01	1.34E-297	cDC2	GRCm39-Il1r1
1.98E-263	6.65921452	0.26	0.00	8.21E-259	cDC2	GRCm39-Cd226
1.32E-260	6.05736983	0.30	0.01	5.49E-256	cDC2	GRCm39-Gm35113
2.06E-254	6.25771376	0.27	0.00	8.53E-250	cDC2	GRCm39-Ehf

bioRxiv preprint doi: https://doi.org/10.64898/2025.12.11.693582; this version posted December 14, 2025. The copyright holder for this preprint (which was not certified by peer review) is the author/funder, who has granted bioRxiv a license to display the preprint in perpetuity. It is made available under aCC-BY 4.0 International license.

0	6.3366805	0.72	0.03	0	IFN-resp	GRCm39-Ifit3
0	5.53060473	0.68	0.03	0	IFN-resp	GRCm39-Oasl2
2.59E-302	6.44811884	0.42	0.01	1.07E-297	IFN-resp	GRCm39-Ifit3b
2.51E-251	4.70583169	0.83	0.06	1.04E-246	IFN-resp	GRCm39-Irf7
6.16E-247	5.62025082	0.76	0.05	2.55E-242	IFN-resp	GRCm39-Isg15
4.01E-240	6.17603993	0.50	0.02	1.66E-235	IFN-resp	GRCm39-Ifit2
7.83E-226	5.25576832	0.53	0.02	3.25E-221	IFN-resp	GRCm39-Zbp1
3.86E-197	4.93797621	0.63	0.04	1.60E-192	IFN-resp	GRCm39-Usp18
1.32E-192	6.11858461	0.39	0.01	5.48E-188	IFN-resp	GRCm39-Ifit1
5.38E-192	4.55435325	0.76	0.07	2.23E-187	IFN-resp	GRCm39-Slfn5
7.24E-187	4.99528459	0.44	0.02	3.00E-182	IFN-resp	GRCm39-Oasl1
8.34E-171	4.4117836	0.56	0.04	3.46E-166	IFN-resp	GRCm39-Mx1
6.90E-150	4.63024551	0.46	0.03	2.86E-145	IFN-resp	GRCm39-Pde7b
5.57E-143	4.54554848	0.30	0.01	2.31E-138	IFN-resp	GRCm39-Gm13822
8.64E-141	4.79986492	0.39	0.02	3.58E-136	IFN-resp	GRCm39-Oas1g
2.34E-140	5.28097535	0.36	0.02	9.69E-136	IFN-resp	GRCm39-Tgtp2
2.66E-138	4.47021931	0.43	0.03	1.10E-133	IFN-resp	GRCm39-Ifi204
9.63E-127	4.48160653	0.52	0.05	3.99E-122	IFN-resp	GRCm39-Ifi47
6.19E-89	4.8206985	0.28	0.02	2.57E-84	IFN-resp	GRCm39-Nlrc5
4.75E-88	4.38026699	0.37	0.03	1.97E-83	IFN-resp	GRCm39-Ifi209
0	11.2588307	1.00	0.00	0	Proliferating	GRCm39-Birc5
0	10.6007107	0.98	0.00	0	Proliferating	GRCm39-Mki67
0	10.9019181	0.88	0.00	0	Proliferating	GRCm39-Prc1
0	10.9503026	0.87	0.00	0	Proliferating	GRCm39-Nusap1
0	15.1652952	0.85	0.00	0	Proliferating	GRCm39-Ube2c
0	10.941932	0.77	0.00	0	Proliferating	GRCm39-Ccna2
0	12.0675414	0.72	0.00	0	Proliferating	GRCm39-Hmmr
0	12.7799322	0.70	0.00	0	Proliferating	GRCm39-Pimreg

bioRxiv preprint doi: https://doi.org/10.64898/2025.12.11.693582; this version posted December 14, 2025. The copyright holder for this preprint (which was not certified by peer review) is the author/funder, who has granted bioRxiv a license to display the preprint in perpetuity. It is made available under aCC-BY 4.0 International license.

0	11.9538644	0.65	0.00	0	Proliferating	GRCm39-Aspm
0	10.8478985	0.63	0.00	0	Proliferating	GRCm39-Pbk
0	10.4727632	0.62	0.00	0	Proliferating	GRCm39-H1f5
0	10.5800527	0.58	0.00	0	Proliferating	GRCm39-Bub1
0	10.7755685	0.50	0.00	0	Proliferating	GRCm39-Ankle1
0	11.7540403	0.50	0.00	0	Proliferating	GRCm39-Kif18b
0	11.8769646	0.42	0.00	0	Proliferating	GRCm39-Cdc25c
0	11.5402759	0.42	0.00	0	Proliferating	GRCm39-H3c3
0	10.536682	0.40	0.00	0	Proliferating	GRCm39-E2f7
0	11.0785587	0.35	0.00	0	Proliferating	GRCm39-Fam83d
0	11.031469	0.30	0.00	0	Proliferating	GRCm39-Sapcd2
0	10.745815	0.25	0.00	0	Proliferating	GRCm39-H2ac22
0	8.29479506	0.89	0.00	0	Neutrophil	GRCm39-Hp
0	13.625857	0.54	0.00	0	Neutrophil	GRCm39-Mmp8
0	8.53852679	0.52	0.00	0	Neutrophil	GRCm39-Ly6a2
0	8.29386748	0.44	0.00	0	Neutrophil	GRCm39-S100a8
0	8.37039651	0.44	0.00	0	Neutrophil	GRCm39-S100a9
0	9.32600374	0.40	0.00	0	Neutrophil	GRCm39-Slfn4
0	18.4454432	0.39	0.00	0	Neutrophil	GRCm39-Retnlg
0	10.6308654	0.39	0.00	0	Neutrophil	GRCm39-Padi4
0	9.52514942	0.37	0.00	0	Neutrophil	GRCm39-Ly6g
0	9.53368561	0.37	0.00	0	Neutrophil	GRCm39-Cd3d
0	12.1699078	0.33	0.00	0	Neutrophil	GRCm39-Bmx
0	12.9338288	0.25	0.00	0	Neutrophil	GRCm39-Il36g
1.51E-263	10.4198043	0.25	0.00	6.28E-259	Neutrophil	GRCm39-F730016J06Rik
1.66E-263	10.2650947	0.25	0.00	6.89E-259	Neutrophil	GRCm39-Gm16556
1.19E-257	9.14713398	0.44	0.01	4.94E-253	Neutrophil	GRCm39-Cxcr2
1.31E-243	9.51706882	0.29	0.00	5.43E-239	Neutrophil	GRCm39-Ankrd22

bioRxiv preprint doi: https://doi.org/10.64898/2025.12.11.693582; this version posted December 14, 2025. The copyright holder for this preprint (which was not certified by peer review) is the author/funder, who has granted bioRxiv a license to display the preprint in perpetuity. It is made available under aCC-BY 4.0 International license.

9.31E-231	8.46292	0.39	0.00	3.86E-226	Neutrophil	GRCm39-Rnf144a
1.91E-210	9.42884049	0.25	0.00	7.93E-206	Neutrophil	GRCm39-Trem1
3.27E-203	9.12287851	0.27	0.00	1.35E-198	Neutrophil	GRCm39-9830107B12Rik
5.29E-195	8.35809077	0.27	0.00	2.19E-190	Neutrophil	GRCm39-Slco4c1
0	7.96655353	0.71	0.01	0	cDC1	GRCm39-Ccser1
0	7.68163066	0.69	0.00	0	cDC1	GRCm39-Ly75
0	8.45800956	0.69	0.01	0	cDC1	GRCm39-Xcr1
0	7.97669841	0.66	0.01	0	cDC1	GRCm39-Strip2
0	8.28893183	0.66	0.01	0	cDC1	GRCm39-Clec9a
0	8.77217353	0.60	0.00	0	cDC1	GRCm39-Zfp366
0	9.76750259	0.54	0.00	0	cDC1	GRCm39-Gcsam
0	8.12004391	0.51	0.00	0	cDC1	GRCm39-Gm36723
0	8.07514578	0.43	0.00	0	cDC1	GRCm39-Htr7
0	11.1136247	0.34	0.00	0	cDC1	GRCm39-Gpr141b
0	8.34805227	0.34	0.00	0	cDC1	GRCm39-Ttc39a
0	11.9120946	0.31	0.00	0	cDC1	GRCm39-Cd207
1.28E-280	9.20243537	0.31	0.00	5.32E-276	cDC1	GRCm39-Gm38410
5.17E-265	8.65998723	0.29	0.00	2.14E-260	cDC1	GRCm39-Clnk
1.49E-200	6.96838111	0.40	0.00	6.17E-196	cDC1	GRCm39-Dbn1
9.80E-181	7.12039346	0.49	0.01	4.06E-176	cDC1	GRCm39-P2ry10
1.07E-143	6.92053972	0.31	0.00	4.45E-139	cDC1	GRCm39-Clec1a
6.18E-111	7.11228595	0.26	0.00	2.56E-106	cDC1	GRCm39-Tnfrsf9
2.49E-108	6.78191968	0.29	0.00	1.03E-103	cDC1	GRCm39-Serpinb6b
4.10E-100	6.93257166	0.54	0.02	1.70E-95	cDC1	GRCm39-Traf1
0	14.5884284	0.96	0.00	0	pDC	GRCm39-Klk1b27
0	17.0784045	0.96	0.00	0	pDC	GRCm39-Klk1
0	12.3937369	0.92	0.00	0	pDC	GRCm39-Gm30605
0	15.6643296	0.92	0.00	0	pDC	GRCm39-Iglc3

bioRxiv preprint doi: https://doi.org/10.64898/2025.12.11.693582; this version posted December 14, 2025. The copyright holder for this preprint (which was not certified by peer review) is the author/funder, who has granted bioRxiv a license to display the preprint in perpetuity. It is made available under aCC-BY 4.0 International license.

0	11.9250733	0.92	0.00	0	pDC	GRCm39-Grm8
0	13.0018169	0.88	0.00	0	pDC	GRCm39-Gm56600
0	14.436582	0.83	0.00	0	pDC	GRCm39-Ly6d
0	12.3032616	0.83	0.00	0	pDC	GRCm39-Pir
0	14.3455247	0.75	0.00	0	pDC	GRCm39-Or2m12
0	12.6493737	0.63	0.00	0	pDC	GRCm39-Mzb1
0	13.1835838	0.58	0.00	0	pDC	GRCm39-Gm12253
0	12.026882	0.54	0.00	0	pDC	GRCm39-Gm14207
0	12.2883929	0.50	0.00	0	pDC	GRCm39-Fyb2
0	12.1087056	0.46	0.00	0	pDC	GRCm39-Tbxa2r
0	13.0033152	0.46	0.00	0	pDC	GRCm39-Gm21762
0	13.2845666	0.42	0.00	0	pDC	GRCm39-Klk4
0	12.4191848	0.42	0.00	0	pDC	GRCm39-Cd8b1
0	13.0426955	0.25	0.00	0	pDC	GRCm39-Igkc
0	11.297292	0.25	0.00	0	pDC	GRCm39-Gm32196
2.31E-279	11.7617988	0.25	0.00	9.56E-275	pDC	GRCm39-Clic5
0	2.06629755	0.73	0.21	0	xMG	GRCh38-MROCKI
2.94E-265	1.94768635	0.68	0.26	1.22E-260	xMG	GRCh38-DHRS9
1.19E-200	2.02693332	0.52	0.15	4.93E-196	xMG	GRCh38-ENSG00000286403
1.33E-184	2.1096339	0.53	0.18	5.49E-180	xMG	GRCh38-APOC2
1.57E-173	2.31207347	0.45	0.12	6.51E-169	xMG	GRCh38-DSCAM
4.54E-170	2.11541724	0.45	0.13	1.88E-165	xMG	GRCh38-ENSG00000285367
2.19E-137	2.2121769	0.38	0.11	9.06E-133	xMG	GRCh38-MMP2
2.36E-121	2.15638494	0.36	0.11	9.80E-117	xMG	GRCh38-TMEM52B
1.29E-117	1.94978991	0.39	0.14	5.34E-113	xMG	GRCh38-FMNL1-DT
5.38E-111	1.97928003	0.34	0.10	2.23E-106	xMG	GRCh38-AZIN2
1.13E-100	1.92833241	0.33	0.10	4.70E-96	xMG	GRCh38-ADAM7-AS1
7.35E-98	1.9997429	0.31	0.09	3.05E-93	xMG	GRCh38-IL18

bioRxiv preprint doi: https://doi.org/10.64898/2025.12.11.693582; this version posted December 14, 2025. The copyright holder for this preprint (which was not certified by peer review) is the author/funder, who has granted bioRxiv a license to display the preprint in perpetuity. It is made available under aCC-BY 4.0 International license.

1.05E-92	2.27685307	0.28	0.07	4.37E-88	xMG	GRCh38-HLA-DRA
5.93E-89	1.96739956	0.30	0.09	2.46E-84	xMG	GRCh38-ENC1
8.02E-87	2.06692989	0.26	0.07	3.33E-82	xMG	GRCh38-ENSG00000290858
4.59E-84	2.17119189	0.25	0.07	1.90E-79	xMG	GRCh38-XACT
2.32E-82	2.11951848	0.26	0.07	9.64E-78	xMG	GRCh38-LINC02381
3.23E-81	2.06234019	0.27	0.08	1.34E-76	xMG	GRCh38-EMID1
8.95E-79	1.9950595	0.31	0.12	3.71E-74	xMG	GRCh38-LILRB4
3.57E-78	1.95637407	0.28	0.09	1.48E-73	xMG	GRCh38-ENSG00000287958
0	6.00768885	0.75	0.03	0	xMG-IFN	GRCh38-IFI44L
0	9.10160988	0.49	0.01	0	xMG-IFN	GRCh38-IFITM1
8.35E-230	5.73749292	0.64	0.04	3.46E-225	xMG-IFN	GRCh38-LY6E
3.64E-187	6.07684928	0.85	0.09	1.51E-182	xMG-IFN	GRCh38-IFI6
1.28E-185	5.23979838	0.63	0.05	5.29E-181	xMG-IFN	GRCh38-IFIT1
2.79E-183	5.25276828	0.74	0.07	1.16E-178	xMG-IFN	GRCh38-ISG15
4.86E-157	4.97621867	0.82	0.10	2.02E-152	xMG-IFN	GRCh38-MX1
1.56E-150	5.46656708	0.32	0.01	6.48E-146	xMG-IFN	GRCh38-MX2
8.49E-139	6.12063826	0.39	0.02	3.52E-134	xMG-IFN	GRCh38-IFI27
6.89E-122	5.17403942	0.48	0.04	2.85E-117	xMG-IFN	GRCh38-IFIT3
3.50E-97	3.89579085	0.45	0.04	1.45E-92	xMG-IFN	GRCh38-OAS2
7.76E-93	4.69985256	0.27	0.02	3.22E-88	xMG-IFN	GRCh38-OASL
6.88E-66	4.18470858	0.32	0.03	2.85E-61	xMG-IFN	GRCh38-OAS3
3.98E-65	3.72511088	0.43	0.06	1.65E-60	xMG-IFN	GRCh38-ODF3B
3.00E-61	3.33155762	0.73	0.20	1.24E-56	xMG-IFN	GRCh38-IRF7
1.04E-55	3.73203821	0.41	0.06	4.31E-51	xMG-IFN	GRCh38-SERPING1
3.88E-55	3.15357218	0.53	0.11	1.61E-50	xMG-IFN	GRCh38-IFI44
8.12E-47	3.74988402	0.51	0.12	3.36E-42	xMG-IFN	GRCh38-IFIT2
6.08E-40	3.08417258	0.49	0.12	2.52E-35	xMG-IFN	GRCh38-HERC5
2.46E-32	3.12180448	0.39	0.09	1.02E-27	xMG-IFN	GRCh38-LGALS3BP

bioRxiv preprint doi: https://doi.org/10.64898/2025.12.11.693582; this version posted December 14, 2025. The copyright holder for this preprint (which was not certified by peer review) is the author/funder, who has granted bioRxiv a license to display the preprint in perpetuity. It is made available under aCC-BY 4.0 International license.

3.76E-282	2.93635771	0.64	0.10	1.56E-277	pre-xMG	GRCh38-HELLS
6.98E-273	4.25738482	0.37	0.02	2.89E-268	pre-xMG	GRCh38-CDCA7
9.79E-264	3.9761682	0.37	0.03	4.06E-259	pre-xMG	GRCh38-GINS2
7.23E-209	3.14591955	0.40	0.04	3.00E-204	pre-xMG	GRCh38-CENPK
2.54E-205	3.00834443	0.49	0.07	1.05E-200	pre-xMG	GRCh38-MCM4
7.85E-188	3.45165183	0.33	0.03	3.26E-183	pre-xMG	GRCh38-CHEK1
2.62E-186	3.51159644	0.30	0.03	1.09E-181	pre-xMG	GRCh38-CDT1
7.44E-184	2.6311589	0.63	0.15	3.08E-179	pre-xMG	GRCh38-MCM3
2.06E-183	3.03314677	0.35	0.04	8.52E-179	pre-xMG	GRCh38-CLSPN
1.46E-151	2.9181721	0.36	0.05	6.06E-147	pre-xMG	GRCh38-ORC6
1.06E-150	3.08664137	0.33	0.04	4.39E-146	pre-xMG	GRCh38-MCM2
1.93E-150	2.63598287	0.48	0.10	7.99E-146	pre-xMG	GRCh38-MCM6
3.02E-149	3.39442539	0.27	0.03	1.25E-144	pre-xMG	GRCh38-DTL
1.17E-130	2.83439747	0.31	0.04	4.83E-126	pre-xMG	GRCh38-RAD51AP1
1.64E-122	3.07133791	0.27	0.03	6.79E-118	pre-xMG	GRCh38-WEE1
1.20E-118	2.6010381	0.34	0.06	4.96E-114	pre-xMG	GRCh38-ZWINT
7.61E-118	3.00440053	0.27	0.04	3.15E-113	pre-xMG	GRCh38-CDC7
3.78E-116	2.65612486	0.33	0.06	1.57E-111	pre-xMG	GRCh38-FEN1
1.65E-109	2.84317776	0.31	0.05	6.85E-105	pre-xMG	GRCh38-WDHD1
1.04E-91	2.65554286	0.25	0.04	4.30E-87	pre-xMG	GRCh38-CENPU
0	5.49295552	0.99	0.05	0	xBAM	GRCh38-RNASE1
0	6.12438364	0.97	0.05	0	xBAM	GRCh38-SELENOP
0	5.29709059	0.86	0.03	0	xBAM	GRCh38-F13A1
0	5.30726284	0.87	0.04	0	xBAM	GRCh38-CD163
0	5.35640789	0.77	0.02	0	xBAM	GRCh38-SCN9A
0	5.9957527	0.74	0.02	0	xBAM	GRCh38-LILRB5
0	5.23356962	0.66	0.02	0	xBAM	GRCh38-CD28
0	5.02876165	0.57	0.02	0	xBAM	GRCh38-TGFBI

bioRxiv preprint doi: https://doi.org/10.64898/2025.12.11.693582; this version posted December 14, 2025. The copyright holder for this preprint (which was not certified by peer review) is the author/funder, who has granted bioRxiv a license to display the preprint in perpetuity. It is made available under aCC-BY 4.0 International license.

0	4.90401111	0.54	0.03	0	xBAM	GRCh38-PMP22
0	5.87762864	0.50	0.01	0	xBAM	GRCh38-CD200R1
0	5.24709438	0.44	0.01	0	xBAM	GRCh38-ENSG00000257060
0	5.1356418	0.42	0.02	0	xBAM	GRCh38-ANTXR2
0	5.64454033	0.31	0.01	0	xBAM	GRCh38-ENSG00000272234
0	5.40795471	0.31	0.01	0	xBAM	GRCh38-TGFBR3
0	5.3259312	0.30	0.01	0	xBAM	GRCh38-ENSG00000258631
0	5.29086671	0.28	0.01	0	xBAM	GRCh38-SIGLEC1
0	4.94807732	0.28	0.01	0	xBAM	GRCh38-ENSG00000253557
0	5.03131921	0.27	0.01	0	xBAM	GRCh38-PID1
1.00E-297	4.92991811	0.27	0.01	4.15E-293	xBAM	GRCh38-SYT1
3.58E-294	5.24910316	0.26	0.01	1.48E-289	xBAM	GRCh38-TMEM37
0	9.12903556	0.66	0.01	0	xProlif	GRCh38-MKI67
0	10.2654841	0.52	0.00	0	xProlif	GRCh38-RRM2
0	9.90929946	0.52	0.00	0	xProlif	GRCh38-ANLN
0	8.6975863	0.50	0.01	0	xProlif	GRCh38-ASPM
0	9.39048304	0.42	0.00	0	xProlif	GRCh38-AURKB
0	8.65263377	0.42	0.00	0	xProlif	GRCh38-UBE2C
0	8.28318928	0.41	0.00	0	xProlif	GRCh38-CKAP2L
0	8.92511724	0.38	0.00	0	xProlif	GRCh38-CCNA2
0	9.53889917	0.36	0.00	0	xProlif	GRCh38-KIF23
0	10.364497	0.32	0.00	0	xProlif	GRCh38-HJURP
0	10.808586	0.32	0.00	0	xProlif	GRCh38-CDCA3
0	10.0758279	0.32	0.00	0	xProlif	GRCh38-KIF18B
0	9.70815919	0.32	0.00	0	xProlif	GRCh38-DLGAP5
0	9.03752173	0.32	0.00	0	xProlif	GRCh38-KIF14
0	8.38245179	0.31	0.00	0	xProlif	GRCh38-CDC25C
0	9.07725179	0.28	0.00	0	xProlif	GRCh38-DEPDC1

bioRxiv preprint doi: https://doi.org/10.64898/2025.12.11.693582; this version posted December 14, 2025. The copyright holder for this preprint (which was not certified by peer review) is the author/funder, who has granted bioRxiv a license to display the preprint in perpetuity. It is made available under aCC-BY 4.0 International license.

0	8.56865038	0.28	0.00	0	xProlif	GRCh38-H3C2
0	8.86739269	0.27	0.00	0	xProlif	GRCh38-PBK
0	10.2768427	0.26	0.00	0	xProlif	GRCh38-E2F7
0	9.1394729	0.26	0.00	0	xProlif	GRCh38-ESPL1

Table S1. Top genes per cell type in scRNAseq dataset

Marker genes per cell type. Prefix in gene name corresponds to genome annotation. GRC = Genome Reference Consortium, GRCm39 = Mus musculus 39, GRCh38 = Homo sapiens 38.

bioRxiv preprint doi: https://doi.org/10.64898/2025.12.11.693582; this version posted December 14, 2025. The copyright holder for this preprint (which was not certified by peer review) is the author/funder, who has granted bioRxiv a license to display the preprint in perpetuity. It is made available under aCC-BY 4.0 International license.

TABLE S2

MURINE					HUMAN				
gene	logFC	pct.1	pct.2	padj	gene	logFC	pct.1	pct.2	padj
Cd36	8.78	0.41	0.00	2.00E-277	CD36	5.68	0.98	0.16	0
Lyve1	8.27	0.45	0.00	1.23E-296	LYVE1	2.63	0.98	0.55	0
Ms4a7	7.25	0.82	0.01	0	MS4A7	1.92	0.85	0.46	0
Dab2	7.12	0.95	0.01	0	DAB2	5.38	1.00	0.53	0
F13a1	7.05	0.94	0.01	0	F13A1	10.97	0.86	0.00	0
Cd163	6.67	0.63	0.00	0	CD163	8.93	0.87	0.01	0
Mrc1	6.31	0.98	0.01	0	MRC1	3.36	0.97	0.49	0
Trps1	6.02	0.50	0.00	0	TRPS1	2.05	0.49	0.20	4.16E-136
Iqgap1	5.48	0.53	0.02	2.05E-304	IQGAP1	4.27	0.68	0.09	0
Itsn1	4.46	0.62	0.05	1.71E-299	ITSN1	4.73	0.75	0.10	0
Tgfbi	4.16	0.84	0.16	0	TGFBI	9.98	0.57	0.00	0
Nrp1	4.01	0.62	0.05	1.37E-304	NRP1	3.48	0.93	0.40	0
Igfbp4	3.72	0.90	0.32	0	IGFBP4	6.99	0.78	0.02	0
Myo5a	3.53	0.67	0.11	7.75E-283	MYO5A	2.29	0.59	0.22	6.71E-216
Stab1	3.34	0.89	0.43	0	STAB1	2.36	0.93	0.65	0
Wwp1	3.23	0.80	0.25	0	WWP1	4.90	0.80	0.13	0
Blvrb	3.16	0.84	0.30	0	BLVRB	3.24	0.83	0.30	0
Mpp1	3.11	0.68	0.16	4.23E-262	MPP1	1.17	0.43	0.27	1.96E-47
Rbpj	3.10	0.75	0.26	0	RBPJ	3.44	0.83	0.37	0
Gas6	3.00	0.90	0.41	0	GAS6	2.15	0.83	0.47	0
Emp3	2.91	0.77	0.27	3.18E-299	EMP3	2.82	0.53	0.10	2.72E-259
Colec12	2.69	0.82	0.37	0	COLEC12	11.07	0.88	0.00	0
Arhgap18	2.35	0.48	0.15	5.36E-100	ARHGAP18	2.03	0.59	0.26	2.16E-194
Eps15	2.25	0.64	0.25	5.67E-161	EPS15	2.59	0.78	0.35	0

bioRxiv preprint doi: https://doi.org/10.64898/2025.12.11.693582; this version posted December 14, 2025. The copyright holder for this preprint (which was not certified by peer review) is the author/funder, who has granted bioRxiv a license to display the preprint in perpetuity. It is made available under aCC-BY 4.0 International license.

Ap2a2	2.06	0.46	0.16	7.44E-82	AP2A2	1.95	0.51	0.21	2.89E-142
Snx3	2.04	0.83	0.48	2.46E-292	SNX3	1.05	0.79	0.68	2.55E-178
Cstb	1.99	0.66	0.32	1.50E-137	CSTB	1.14	0.68	0.51	7.83E-119
Txnip	1.88	0.90	0.66	2.44E-293	TXNIP	1.96	0.79	0.47	0
Selenop	1.79	1.00	0.99	0	SELENOP	8.82	0.97	0.03	0
Tle5	1.77	0.76	0.44	1.61E-201	TLE5	1.01	0.42	0.25	1.21E-30
Eps8	1.73	0.43	0.17	4.03E-54	EPS8	1.87	0.69	0.37	4.05E-248
Ap2s1	1.71	0.63	0.32	8.90E-107	AP2S1	1.10	0.64	0.44	9.14E-87
Crtap	1.68	0.42	0.17	4.80E-52	CRTAP	1.11	0.53	0.35	2.74E-62
Blvra	1.66	0.50	0.23	3.61E-63	BLVRA	5.12	0.53	0.03	0
Slc9a9	1.60	0.86	0.74	2.33E-219	SLC9A9	1.28	0.96	0.93	0
Hint1	1.57	0.81	0.54	1.80E-184	HINT1	1.07	0.71	0.54	3.53E-104
Bri3	1.56	0.83	0.61	8.27E-207	BRI3	1.22	0.88	0.77	4.13E-305
Cltc	1.52	0.91	0.79	7.41E-269	CLTC	1.54	0.85	0.63	0
Arpc5	1.52	0.77	0.51	1.91E-170	ARPC5	1.11	0.81	0.69	1.36E-210
Snx6	1.36	0.73	0.53	8.77E-98	SNX6	2.72	0.90	0.48	0
Bloc1s1	1.24	0.52	0.30	9.31E-41	BLOC1S1	1.20	0.54	0.34	7.49E-65
Npl	1.16	0.41	0.29	8.08E-27	NPL	1.77	0.72	0.42	1.87E-242
Sdcbp	1.08	0.78	0.62	3.66E-105	SDCBP	1.09	0.67	0.50	1.01E-112
Rpl23	1.06	0.98	0.99	7.65E-191	RPL23	1.12	0.91	0.80	6.53E-268
Rps16	1.05	0.98	0.98	1.95E-194	RPS16	1.14	0.95	0.92	0
Rps14	1.03	0.97	0.97	7.33E-186	RPS14	1.19	0.98	0.98	0
Ly86	-1.04	0.89	0.99	4.67E-230	LY86	-2.19	0.51	0.95	0
Cd53	-1.06	0.72	0.94	4.03E-136	CD53	-1.68	0.66	0.97	0
Slc4a7	-1.09	0.30	0.49	5.33E-32	SLC4A7	-2.08	0.35	0.83	0
Pmepa1	-1.09	0.57	0.97	1.22E-212	PMEPA1	-2.72	0.22	0.73	0
Arhgap22	-1.14	0.25	0.49	8.76E-37	ARHGAP22	-2.98	0.08	0.40	1.40E-151
Saraf	-1.14	0.43	0.76	1.63E-81	SARAF	-1.21	0.67	0.94	3.07E-269

bioRxiv preprint doi: https://doi.org/10.64898/2025.12.11.693582; this version posted December 14, 2025. The copyright holder for this preprint (which was not certified by peer review) is the author/funder, who has granted bioRxiv a license to display the preprint in perpetuity. It is made available under aCC-BY 4.0 International license.

Laptm5	-1.18	0.98	1.00	0	LAPTM5	-2.22	0.80	1.00	0
Sh3kbp1	-1.19	0.42	0.67	3.74E-59	SH3KBP1	-2.09	0.27	0.69	1.51E-235
Hps3	-1.21	0.31	0.53	5.40E-42	HPS3	-2.44	0.12	0.45	9.53E-142
Slco2b1	-1.25	0.69	0.94	1.24E-169	SLCO2B1	-1.49	0.71	0.96	0
Hivep3	-1.30	0.32	0.63	2.23E-63	HIVEP3	-2.19	0.17	0.56	1.49E-177
Eml4	-1.30	0.29	0.52	4.04E-47	EML4	-1.99	0.33	0.74	1.73E-244
Ckb	-1.33	0.70	0.97	5.66E-227	CKB	-9.98	0.00	0.82	0
Lpin2	-1.34	0.36	0.69	1.38E-78	LPIN2	-1.08	0.54	0.81	5.78E-132
Gmip	-1.35	0.40	0.71	3.17E-83	GMIP	-1.38	0.23	0.46	6.05E-71
Prex1	-1.37	0.31	0.60	1.28E-63	PREX1	-1.04	0.43	0.68	3.49E-89
Bhlhe41	-1.38	0.24	0.49	5.82E-45	BHLHE41	-4.09	0.14	0.84	0
Blnk	-1.43	0.47	0.82	1.77E-124	BLNK	-1.45	0.42	0.75	6.18E-166
Atp2c1	-1.44	0.25	0.47	4.01E-47	ATP2C1	-2.31	0.21	0.63	2.69E-221
Otulinl	-1.53	0.63	0.95	1.34E-238	OTULINL	-2.02	0.48	0.90	0
Trem2	-1.56	0.80	0.99	0	TREM2	-3.53	0.30	0.99	0
Cyrib	-1.56	0.62	0.94	1.36E-237	CYRIB	-1.13	0.60	0.86	1.97E-178
Ankrd44	-1.57	0.39	0.74	3.15E-111	ANKRD44	-2.47	0.41	0.92	0
Evl	-1.57	0.32	0.67	5.36E-92	EVL	-1.19	0.62	0.90	1.07E-211
Susd3	-1.65	0.35	0.76	4.14E-128	SUSD3	-1.50	0.30	0.59	5.19E-114
Mef2a	-1.70	0.66	0.98	0	MEF2A	-1.34	0.83	0.99	0
Entpd1	-1.70	0.54	0.92	4.33E-235	ENTPD1	-2.37	0.48	0.96	0
Daglb	-1.78	0.42	0.85	3.32E-183	DAGLB	-1.26	0.20	0.41	1.07E-54
Tcirg1	-1.91	0.42	0.85	3.59E-198	TCIRG1	-1.37	0.25	0.51	3.69E-86
Samsn1	-1.92	0.21	0.55	2.35E-83	SAMSN1	-1.85	0.63	0.95	0
Slc8a1	-1.97	0.45	0.92	8.40E-259	SLC8A1	-1.77	0.47	0.91	0
Inpp5d	-1.98	0.57	0.96	0	INPP5D	-1.29	0.56	0.85	1.51E-202
Lrrk1	-2.03	0.17	0.48	1.18E-76	LRRK1	-1.63	0.39	0.76	7.04E-193
Sting1	-2.08	0.42	0.87	1.26E-229	STING1	-2.58	0.22	0.68	3.44E-273

bioRxiv preprint doi: https://doi.org/10.64898/2025.12.11.693582; this version posted December 14, 2025. The copyright holder for this preprint (which was not certified by peer review) is the author/funder, who has granted bioRxiv a license to display the preprint in perpetuity. It is made available under aCC-BY 4.0 International license.

Skil	-2.11	0.22	0.60	1.63E-110	SKIL	-2.48	0.17	0.57	6.76E-199
Rhob	-2.12	0.57	0.96	0	RHOB	-2.04	0.28	0.65	2.05E-179
Dock10	-2.12	0.45	0.90	3.41E-259	DOCK10	-2.38	0.33	0.84	0
Apbb1ip	-2.22	0.55	0.97	0	APBB1IP	-2.89	0.57	0.99	0
Cx3cr1	-2.26	0.79	1.00	0	CX3CR1	-3.80	0.55	1.00	0
Cd81	-2.33	0.62	1.00	0	CD81	-1.71	0.86	1.00	0
Vsir	-2.34	0.64	0.99	0	VSIR	-4.12	0.26	0.97	0
Specc1	-2.48	0.20	0.61	2.05E-130	SPECC1	-1.10	0.41	0.69	2.69E-99
Basp1	-2.49	0.49	0.98	0	BASP1	-7.69	0.01	0.48	0
Ptgs1	-2.50	0.46	0.96	0	PTGS1	-2.91	0.16	0.64	2.31E-284
Slc29a3	-2.50	0.32	0.81	3.76E-235	SLC29A3	-2.28	0.23	0.65	8.81E-228
P2ry13	-2.51	0.37	0.90	0	P2RY13	-3.65	0.38	0.98	0
Pald1	-2.52	0.18	0.61	3.05E-137	PALD1	-2.04	0.26	0.66	4.23E-210
Serpinf1	-2.58	0.24	0.62	8.68E-113	SERPINF1	-3.16	0.15	0.69	0
Ywhah	-2.62	0.46	0.96	0	YWHAH	-3.09	0.38	0.94	0
Il6st	-2.63	0.19	0.59	6.39E-137	IL6ST	-1.17	0.65	0.90	8.58E-191
Fmnl3	-2.70	0.23	0.75	9.03E-216	FMNL3	-1.32	0.38	0.69	3.13E-131
Csf3r	-2.81	0.25	0.78	9.29E-244	CSF3R	-2.52	0.42	0.93	0
Tia1	-2.85	0.10	0.40	6.85E-89	TIA1	-1.02	0.27	0.46	2.02E-43
Sema4d	-2.86	0.15	0.61	3.37E-157	SEMA4D	-1.80	0.22	0.55	3.83E-141
Chd7	-2.94	0.16	0.60	1.95E-155	CHD7	-1.54	0.36	0.70	1.91E-162
Gpr34	-2.97	0.60	1.00	0	GPR34	-1.59	0.73	0.97	0
Dock4	-2.98	0.39	0.95	0	DOCK4	-2.47	0.57	0.99	0
Rasal3	-3.14	0.11	0.53	8.63E-138	RASAL3	-1.76	0.24	0.58	1.49E-140
Dst	-3.19	0.16	0.68	2.24E-206	DST	-1.92	0.19	0.47	7.47E-114
Epb41l2	-3.33	0.32	0.93	0	EPB41L2	-2.50	0.22	0.66	6.96E-250
Bin2	-3.39	0.27	0.91	0	BIN2	-2.24	0.39	0.85	0
Lpcat2	-3.49	0.39	0.99	0	LPCAT2	-2.36	0.32	0.80	0

bioRxiv preprint doi: https://doi.org/10.64898/2025.12.11.693582; this version posted December 14, 2025. The copyright holder for this preprint (which was not certified by peer review) is the author/funder, who has granted bioRxiv a license to display the preprint in perpetuity. It is made available under aCC-BY 4.0 International license.

Pde3b	-3.51	0.32	0.96	0	PDE3B	-1.84	0.18	0.46	2.25E-101
Mertk	-3.57	0.25	0.93	0	MERTK	-2.30	0.43	0.89	0
Abi3	-3.72	0.19	0.90	0	ABI3	-5.84	0.02	0.58	0
Plxdc2	-4.43	0.20	0.99	0	PLXDC2	-2.57	0.68	1.00	0
Olfml3	-4.54	0.17	0.98	0	OLFML3	-7.90	0.02	0.98	0
Frmd4a	-4.57	0.15	0.92	0	FRMD4A	-2.17	0.65	0.99	0
P2ry12	-4.98	0.52	1.00	0	P2RY12	-4.66	0.46	1.00	0
Sgk1	-5.31	0.06	0.70	1.08E-290	SGK1	-1.58	0.22	0.45	3.46E-73
Selplg	-5.42	0.39	0.99	0	SELPLG	-5.37	0.14	0.95	0
Tmem119	-6.02	0.13	0.99	0	TMEM119	-6.78	0.03	0.84	0
Gal3st4	-6.98	0.02	0.84	0	GAL3ST4	-4.32	0.18	0.97	0
Lhfpl2	-7.08	0.02	0.66	2.55E-305	LHFPL2	-3.09	0.18	0.72	0
Med12l	-7.25	0.01	0.63	5.51E-288	MED12L	-3.86	0.06	0.50	4.20E-247
Slc1a3	-8.95	0.00	0.63	1.78E-295	SLC1A3	-1.37	0.41	0.71	4.13E-116
Sall1	-9.15	0.00	0.84	0	SALL1	-6.41	0.01	0.54	0

Table S2. Conserved BAM versus microglia signature across human and murine origin

Gene set distinguishing BAMs from microglia of both human and murine origin. Genes were filtered based on a logFC threshold of 1 (corresponding to 2-fold enrichment or de-enrichment in BAMs), a min.pct of 0.4 (corresponding to at least 40% expression), and orthology verification across species.

bioRxiv preprint doi: https://doi.org/10.64898/2025.12.11.693582; this version posted December 14, 2025. The copyright holder for this preprint (which was not certified by peer review) is the author/funder, who has granted bioRxiv a license to display the preprint in perpetuity. It is made available under aCC-BY 4.0 International license.

TABLE S3

Antigen	Species reactivity	Fluorophore	Clone	Manufacturer	Cat #	Dilution	Note
CD45	mouse	PE-Cy5.5	30-F11	ThermoFisher	35-0451-82	500	Used 1:200 for FEAST
CD45	mouse	BV605	30-F11	BioLegend	103155	500	
GR-1	mouse	BUV805	RB6-8C5	BD Biosciences	741920	100	
CD68	mouse	APC-Cy7	FA-11	BioLegend	137024	400	
CD206	mouse	PE-Cy7	C068C2	BioLegend	141720	200	
CD206	mouse	BV421	C068C2	BioLegend	141717	200	
CX3CR1	mouse	BV711	SA011F11	BioLegend	149031	800	
MHC-II	mouse	BV785	M5/114.15.2	BioLegend	107645	200	
MBP	mouse/human/rat	AF647	P82H9	BioLegend	850910	200	
CD38	mouse	BUV395	90/CD38	BD Biosciences	740245	200	
CD64	mouse	PE-Cy7	X54-5/7.1	BioLegend	139313	100	
CD11b	mouse/human/rhesus	BV605	M1/70	BioLegend	101237	200	
CD45	human	AF488	HI30	BioLegend	304017	200	
CD45	human	BV421	HI30	BioLegend	304032	200	
CD206	human	PE	15-2	BioLegend	321105	200	
CD206	human	AF647	15-2	BioLegend	321116	200	

Table S3. Flow cytometry antibodies for distinguishing BAMs and xBAMs

Recommended antibodies and dilutions for flow cytometry panel to identify BAMs and xBAMs in chimeric brains. For live cell preparations, we recommend identifying BAMs as muCD45^{+}/ GR1^{-}/ CD11b^{+}CD64^{+}/ CX3CR1^{int}/CD38^{+} and xBAMs as huCD45^{+}/huCD206^{hi}. For fixed cell FEAST preparations, we recommend identifying BAMs as muCD45^{+}/GR1^{-}/CD68^{+}/CX3CR1^{int}/CD38^{+} (note: the panel is adjusted due to epitope loss following the fixed-cell protocol). The xBAM gating with huCD45^{+}/huCD206^{hi} is compatible with the fixed cell FEAST protocol and does not require adjustment. See **Figure S3**.
